# Familial Risk of Hashimoto's Thyroiditis in a Large Genealogical Database

**DOI:** 10.1210/clinem/dgaf251

**Published:** 2025-04-28

**Authors:** Melissa Bujnis, Kelsey DeSalvo, Deborah W Neklason, Michael J Madsen, Lynn B Jorde

**Affiliations:** Department of Human Genetics, University of Utah, Salt Lake City, UT 84112, USA; Department of Internal Medicine, Division of Endocrinology, Intermountain Health, Salt Lake City, UT 84107, USA; Department of Internal Medicine, Division of Epidemiology, University of Utah, Salt Lake City, UT 84112, USA; Utah Population Database, Huntsman Cancer Institute, University of Utah, Salt Lake City, UT 84112, USA; Department of Human Genetics, University of Utah, Salt Lake City, UT 84112, USA

**Keywords:** Hashimoto's thyroiditis, HT, UPDB, autoimmune hypothyroidism

## Abstract

**Context:**

Autoimmune hypothyroidism, commonly known as Hashimoto thyroiditis (HT), is an autoimmune thyroid disorder affecting approximately 5% of the US population. Previous relative risk studies have suggested that first-degree relatives of individuals with HT are at ∼4.5 to 32 times higher risk for developing HT than the general population. Twin studies estimate high heritability for the development of HT (∼65%).

**Objective:**

In this study, we aimed to better estimate the HT relative risk in first-, second-, and third-degree relatives in the Utah Population Database.

**Methods:**

From the Utah Population Database, a total of 92 405 HT probands and 184 810 matched controls were identified, with 2 960 650 relatives of HT probands and 5 730 159 relatives of controls, making this the largest relative risk study of HT.

**Results:**

Females with HT in this cohort were 2.71-fold more common than males. The odds ratio (OR) of HT in the first-degree relatives of affected individuals is 1.77 (95% CI, 1.74-1.80). The OR of HT in second-degree relatives is 1.23 (95% CI, 1.22-1.27) and 1.11 (95% CI, 1.10-1.12) in third-degree relatives of HT probands. We also identified an increased OR of spouses to develop HT of 1.50 for husbands of affected wives (95% CI, 1.39-1.61) and 1.58 for wives of affected husbands (95% CI, 1.47-1.70), suggesting a significant environmental component contributing to HT development.

**Conclusion:**

This is the first study to estimate an increased risk of HT for second- and third-degree relatives, who are less likely to share common environments than first-degree relatives.

Autoimmune hypothyroidism, also known as Hashimoto thyroiditis (HT), is a common autoimmune thyroid disorder affecting approximately 5% of the US population, with similar estimates globally (1-3). HT is characterized by inadequate thyroid hormone production (hypothyroidism) resulting from autoimmune destruction of the thyroid gland, usually diagnosed by the presence of antithyroid antibodies (ie, anti-thyroperoxidase and anti-thyroglobulin). Thyroid hormones influence almost every cell in the body (4-6). Therefore, hypothyroidism manifests through a spectrum of symptoms, including fatigue, weight gain, depression, infertility, and cold intolerance, among others (4-6). Individuals with hypothyroidism also have an increased risk of hypertension, dyslipidemia (high low-density lipoprotein cholesterol), type 2 diabetes, cardiovascular disease, and stroke, resulting in a decreased quality of life and an overall increase in mortality (4-6). HT presents an added complication beyond hypothyroidism because of the sustained autoimmune response targeting the thyroid, including an increased risk of developing other autoimmune diseases and continued symptoms of hypothyroidism while on treatment (7, 8).

Although nongenetic environmental factors such as iodine intake, infection, and medications contribute to HT pathogenesis, evidence supports a substantial genetic component in hypothyroidism susceptibility (4, 9). Twin studies estimate the heritability of HT as 65% to 75% (10, 11). Additionally, previous studies have shown that relatives of HT cases are at higher risk for developing HT than relatives of unaffected controls, supporting the influence of genetic factors in disease development (12-14). Previous studies of the relative risk (RR) of HT have been limited to first-degree relatives (FDRs) and, until recently, very small sample sizes (12-14). The magnitude of the FDR RR estimates across studies ranges widely from 4.5 to 32 (12-16). Familial risk studies are fundamental for understanding the heritability of HT and the role genetics plays in its development. However, a more precise RR estimate is needed to better understand the genetic contribution to HT development.

The Utah Population Database (UPDB) offers a unique opportunity to address the limitations of previous studies by providing comprehensive genealogical and medical records for a large population (17-19). Leveraging this rich resource, we aimed to estimate the odds ratio (OR) for HT across different degrees of relatedness, shedding light on the genetic predisposition to this common disease. Specifically, we sought to quantify the risk of HT, including detailed FDR risk, and more distant relatives, including second- and third-degree relatives (SDR, TDR). The UPDB has been instrumental in understanding the genetics of coronary artery disease and breast cancer, among many other diseases and traits (20, 21). We aim to provide a better understanding of the familial risk of HT using this powerful genealogical resource linked to medical records.

## Methods

### Utah Population Database

The study employs a population-based retrospective case-control design using data from the UPDB. The UPDB contains computerized records for the state of Utah that have been linked to statewide medical, genealogical, and demographic data (17, 18, 22, 23). The database includes 11 million individuals with at least 1 health record and 4.5 million who are part of a 3-generation pedigree. We used data sourced from electronic health records (EHR) from 1996 to 2021 from the University of Utah Health and Intermountain Health (IH) electronic data warehouse, statewide ambulatory care and inpatient data, driver license information, birth certificates, death certificates, and the Utah Cancer Registry. Consanguinity estimates for individuals in the UPDB are very low and similar to those of other US populations (19, 24). The UPDB characterizes race and ethnicity by any self-reported document from individuals’ records. Race was classified broadly into White and non-White (including American Indian or Alaska Native, Asian, Native Hawaiian or Other Pacific Islander, Black or African American, Multiple Races, Unknown, and not classified).

### Ethics Approval

This study has been approved by the IH and University of Utah institutional review boards (IRB) and the Resource for Genetic and Epidemiological Research (IRB_00123835, IRB_1051839). The Resource for Genetic and Epidemiological Research is the regulatory body for UPDB data use (25). The research study utilized de-deidentified data, ensuring the anonymity of all subjects involved. A waiver of consent was granted given the absence of identifiable information.

### HT Cases

**Figure 1.**
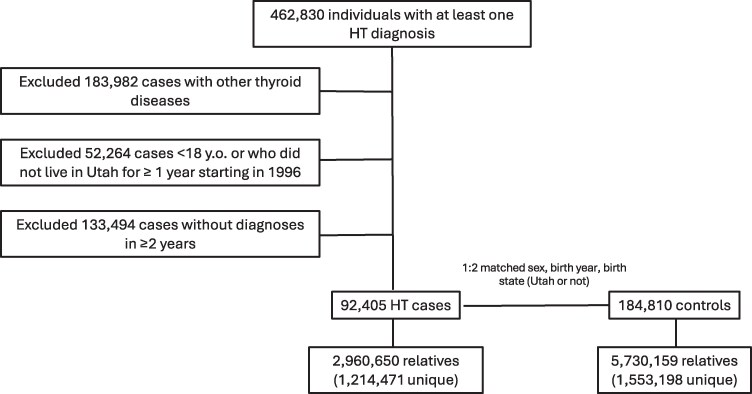
HT familial risk study design. Cases and controls were filtered to exclude nonautoimmune causes of hypothyroidism, individuals without a diagnosis in 2 different years to ensure true HT cases, and 2 matched controls per case.

University of Utah Health and IH EHR were searched to identify patients over the age of 18 years with HT, classified by International Classification of Disease 9th and 10th revision (ICD9 and ICD10) records from 1996 through 2021 (Supplementary Table S1) (26). By using ICD9/10 codes 245.2/E06.3 (autoimmune thyroiditis) and 244.9/E03.9 (hypothyroidism, unspecified) from at least 2 diagnostic records in 2 separate years to identify HT cases, we were able to exclude those who were only tested for HT but never diagnosed with HT. ICD9/10 codes for other causes of HT, including autoimmune hyperthyroidism (Graves’ disease), were used to exclude hypothyroid individuals who are unlikely to have HT (Supplementary Table S1) (26). A total of 226 584 cases were identified, and following the exclusion of other thyroid conditions, the requirement of at least 3 relatives in the UPDB, and age greater than 18 years, 92 405 probands were selected as HT cases (Fig. 1). Cases included individuals with diagnostic ages ranging from ages 18 through 89 years and birth years between 1895 and 2005. Additional information about the proband demographics can be found in Table 1. A total of 2 960 650 total (1 214 471 unique) relatives of HT probands were identified, including 359 144 FDR, 821 765 SDR, and 1 779 741 TDR. FDR includes parents, children, and siblings. SDR includes grandparents and grandchildren, aunts and uncles, nephews and nieces, and half-siblings. TDR includes, but is not limited to, first cousins, great aunts and uncles, grand nephews and grand nieces, great-grandparents, and great-grandchildren. Relatives can occur in multiple pedigrees. Additional information about the relatives’ demographics can be found in Supplementary Tables S2-9 (26).

**Table 1.** Proband demographics

Characteristic	Case, N = 92 405*^a^*	Control, N = 184 810*^a^*	*P* value*^b^*
Sex			>.9
Female	67 549 (73%)	135 098 (73%)	
Male	24 856 (27%)	49 712 (27%)	
Birth year			>.9
1906-1919	4736 (5.1%)	9489 (5.1%)	
1920-1939	25 171 (27%)	50 330 (27%)	
1940-1959	34 446 (37%)	68 887 (37%)	
1960-1979	20 027 (22%)	40 054 (22%)	
1980-2004	8022 (8.7%)	16 044 (8.7%)	
Born in Utah			>.9
N	42 775 (46%)	85 550 (46%)	
Y	49 630 (54%)	99 260 (54%)	
Pedigree size (matching)			>.9
0-4	30 253 (33%)	60 506 (33%)	
5-9	4596 (5.0%)	9192 (5.0%)	
10+	57 556 (62%)	115 112 (62%)	
Maximum degree of relative			>.9
0	20 718 (22%)	41 436 (22%)	
1	7554 (8.2%)	15 108 (8.2%)	
2	7247 (7.8%)	14 494 (7.8%)	
3	56 886 (62%)	113 772 (62%)	
White			<.001
Yes	88 427 (96%)	166 293 (90%)	
No	3978 (4.3%)	18 517 (10%)	
Ethnicity			<.001
Hispanic	6050 (6.5%)	15 715 (8.5%)	
Non-Hispanic	83 359 (90%)	152 063 (82%)	
Unknown	2996 (3.2%)	17 032 (9.2%)	

Case and control proband demographics, including sex, birth year, born in Utah, maximum degree of relatives, race, and ethnicity.

^*a*^n (%).

^*b*^Pearson's chi-squared test.

### Population Controls

Controls were randomly selected and matched to cases by gender, birth year ±2.5 years, pedigree size and quality (the most distant eligible relative), and birth state (Utah vs non-Utah). Controls were also excluded for other causes of hypothyroidism (see Supplementary Table S1 for ICD9/10 exclusionary codes) (26). Two unique controls per case were used for a total of 184 810 controls (see Table 1 for details). There were 5 730 159 relatives of controls (1 553 198 unique individuals) used for OR estimation, including 707 990 FDR, 1 594 392 SDR, and 3 427 777 TDR, allowing individuals to be counted in multiple pedigrees (see Supplementary Tables S2-9 for details) (26).

### Statistical Analysis

We estimated OR and constructed 95% CIs using mixed-effects logistic regression models. In all models, the dependent variable was whether the relative of the case index or control index had HT or not. The independent variables included the case or control status of the index person and the sex of the relative, as applicable. Models were additionally clustered on the index case. Separate mixed-effects logistic regression models were performed to compare relatives of cases to relatives of controls for FDR, SDR, and TDR. These models simultaneously estimate the OR of HT attributable to being a relative of a case and the OR of HT attributable to being female vs being male (Table 2). To explore the possibility of confounding, these models were also run with stratification by race and ethnicity, and by median age. Within the FDR, we performed separate analyses stratified by relationship type to further explore specific familial patterns of risk. For example, we compared HT in sons of fathers with HT to HT in sons of fathers without HT.

**Table 2.** Familial risk of HT, adjusting for sex, for first-, second-, and third-degree relatives

Relation to index	N relatives cases	N relatives controls	OR relative*^a^*	95% CI	*P* value	OR female*^b^*	95% CI	*P* value
FDR	381 575	756 341	1.77	1.74-1.80	**<**.**001**	2.50	2.45-2.55	**<**.**001**
SDR	974 409	189 0743	1.23	1.22-1.25	**<**.**001**	2.63	2.59-2.67	**<**.**001**
TDR	2 288 120	4 372 062	1.11	1.10-1.12	**<**.**001**	2.46	2.44-2.49	**<**.**001**

Significant *P* values are in bold.

Abbreviations: FDR, first-degree relative; OR, odds ratio; SDR, second-degree relative; TDR, third-degree relative.

^*a*^Risk attributable to being a relative of a case compared to being a relative of a control.

^*b*^Risk attributable to being female compared to male.

### Prevalence Estimate of HT

The prevalence of HT was estimated for the Utah population in 2020. For this estimate, we counted all individuals in our sample population with medical records in Utah for 2020 and a first date of residence in Utah before or during 2020 (N = 2 103 271). A single diagnosis prevalence was calculated by counting all those in the at-risk population who had at least 1 HT diagnosis in 2020 or a prior year (N = 123 839). A confirmed diagnosis prevalence was calculated by counting all those in the single diagnosis cohort with at least 1 HT diagnosis in a different year (N = 56 322).

## Figures and Tables

**Table 3.** Specific first-degree risk

Proband (with HT)	Relative	Odds ratio	95% CI	*P* value
Mother	Daughter	1.83	1.74-1.92	**<**.**001**
Mother	Son	1.88	1.74-2.03	**<**.**001**
Father	Daughter	1.80	1.68-1.93	**<**.**001**
Father	Son	2.36	2.09-2.65	**<**.**001**
Female*^a^*	Sister	1.76	1.70-1.83	**<**.**001**
Female*^a^*	Brother	1.70	1.61-1.80	**<**.**001**
Male*^a^*	Sister	1.59	1.50-1.68	**<**.**001**
Male*^a^*	Brother	2.14	1.99-2.31	**<**.**001**
Husband	Wife	1.50	1.39-1.61	**<**.**001**
Wife	Husband	1.58	1.47-1.70	**<**.**001**

Odds ratio of HT among FDR and between spouses, stratified by relationship type.

Significant *P* values are in bold.

Abbreviations: FDR, first-degree relative; HT, Hashimoto thyroiditis.

^*a*^Any FDR of this sex.

## Data Availability

Restrictions apply to the availability of the data analyzed during this study to preserve patient confidentiality. Access to UPDB data is controlled through the Resource for Genetic and Epidemiologic Research (https://uofuhealth.utah.edu/huntsman/utah-population-database/data/access).

## Results

We identified 92 405 HT probands and 184 810 age- and sex-matched controls (1:2) in UPDB born before 2006 using diagnostic EHRs from 1996 to 2021. Table 1 and Supplementary Table S1 summarize the demographic characteristics of the HT cases and matched controls (26). In our cohort, HT was more prevalent in females (73%) than males (27%), similar to previous reports (27-29). A prevalence estimate of HT was also calculated for the year 2020 using our total sample population of cases, controls, and their relatives (N = 2 103 271). A prevalence rate of 5.89% was determined for HT with at least 1 HT diagnosis in 2020 or a prior year (N = 123 839) and 2.68% for those with confirmed diagnoses, classified by at least 2 HT diagnoses in a different year (N = 56 322).

More than 62% of the cases and controls had more than 10 relatives in the UPDB and information for FDR, SDR, and TDR, for a total of 2 960 650 relatives of HT probands and 5 730 159 relatives of controls (relatives can be duplicate individuals belonging to more than 1 family cluster), resulting in large pedigrees that facilitate more accurate RR estimation (Supplementary Table S2) (26). A significantly heightened OR of HT was observed in FDR, SDR, and TDR of HT probands (Table 2). HT occurred in 45 852 (3.8%) unique proband relatives (not counting duplicates) and 51 470 (3.3%) control relatives (*P* < .001). Among those with an affected FDR, there was an absolute increase of 3.0% compared to controls (7.3%, vs 4.3%, *P* < .001), resulting in an OR of HT of 1.77 (95% CI, 1.74-1.80) among all FDRs. The OR in SDRs was 1.25 (1.23-1.27) and 1.11 (1.10-1.12) in TDR of HT probands. In each of these, the female relatives were at an additional risk between 2.2 to 2.6 times greater for developing HT than the overall relative estimates (Table 2). For example, female FDR of HT cases have an OR of 4.42 (1.77 × 2.50). Additionally, when separated into broad race and ethnicity categories of white and non-white, we identified an increased OR across all non-White relatives (Supplementary Table S10) (26). Odds ratios were similar when stratified by birth year of relatives before and after 1974 (Supplementary Table S11) (26). However, the median age of diagnosis for all HT cases (probands and relatives) has decreased over time, from approximately 70 years of age in 1995 to 47 years of age in 2021 (Supplementary Table S12) (26).

A closer examination of FDRs using subgroups of FDRs showed an OR of HT of 1.91 (1.84-1.97) in children of affected parents, a 3.14 (3.02-3.26) OR for female children, and 1.78 (1.73-1.82) in siblings, with an additional 2.38 (2.32-2.45) OR for female siblings of HT probands (Supplementary Table S13) (26). Further broken down, we compared each pair to matched controls (eg, affected mother-son pairs compared to control mother-son pairs), to isolate sex-specific risk patterns (Table 3). The highest risk of HT was observed in the sons of affected fathers compared to sons of unaffected fathers, with an OR of 2.36 (2.10-2.65), and the lowest risk in brother-sister pairs, with an OR of 1.58 (1.49-1.66) (Table 3). The OR of a parent with HT to the child, if the parent is the mother, is 1.83 (1.75-1.92) and 1.90 (1.76-2.05) in daughters and sons, respectively (Table 3). The father-daughter OR of HT is 1.80 (1.67-1.93). In sibling pairs, the OR from a female to a sister is 1.75 (1.69-1.82) and 1.71 (1.62-1.80) to a brother. If the proband is male, the ORs to his sister and brother are 1.58 (1.49-1.66) and 2.14 (1.99-2.31), respectively (Table 3). Spousal OR was also assessed to estimate shared environmental effects. The OR for HT to a wife of a husband with HT was 1.58 (1.53-1.64) and 1.50 (1.39, 1.61) for the reverse. Although these ORs are somewhat lower than those of FDR pairs, they suggest a significant shared environment effect of HT in spouses.

## Discussion

In this study, we used the UPDB to explore the familial risk of HT to provide insights into the genetics of this condition. Our findings indicate a statistically significant increase in the OR of HT among FDR, SDR, and TDR of individuals with HT compared to controls (ORs: 1.73, 1.22, and 1.11, respectively). This is consistent with the substantial heritability of HT, consistent with previous reports highlighting the role of genetic predisposition in the etiology of the disease (10, 12, 14). Our results showed that females were disproportionately affected, with 2.7 times as many female cases as males, resulting in an average 2.54-fold increased OR for females in addition to the combined (across all degrees of relationship) OR, further supporting the known increased prevalence of HT in females.

The observed FDR OR of 1.73 (1.70-1.76) in our study was lower than estimates from 2 recent studies, which reported FDR risk estimates between 4.75 and 6.45 (12, 14). This difference may stem from variations in study design, population characteristics, or methodological approaches to estimating familial risk. Kim et al estimated incidence rate ratios using Korean National Health Insurance records, tracking individuals with and without HT and calculating incidence rates for FDRs based on their years at risk during the study period (12). In contrast, we leverage population and familial data to construct multigenerational pedigrees and derive an OR from probands and their relatives. Thomsen et al used the Swedish Hospital Discharge and Outpatient Registers to estimate familial standardized incidence ratios for FDRs with HT, comparing observed cases to expected population prevalence rather than using a control group (14). Additionally, previous familial risk estimates were based on relatively small pedigrees limited to parents, offspring, and siblings, whereas the UPDB enables us to integrate precise genealogical records with EHR data to construct large, unbiased pedigrees, extending risk assessments to more distant relatives. Differences in diagnostic criteria likely contribute as well, as Korean and Swedish clinics more rigorously utilize antibody testing and ultrasound compared to US clinical practices (12, 14, 30).

Our findings also highlight the role of environmental factors in HT risk, as demonstrated by the elevated ORs observed in spouses. This suggests that shared environmental exposures, such as iodine intake, smoking, and infections, may contribute to HT development (1, 31, 32). Additionally, the elevated affected male to brother and affected father to son ORs are consistent with the sex-specific multifactorial threshold model, which predicts that relatives of the less commonly affected sex (males in this case) are at greater risk because the liability threshold (ie, the number of predisposing genetic or environmental factors) is higher for the less commonly affected sex (33). This pattern could also reflect in part a greater contribution of nongenetic factors in females, such as shared environmental or hormonal influences (34-36). Furthermore, the observation of increased OR in relatives of non-White individuals may result from a difference in environment or genetics. It is not possible to disentangle the proportion of risk due to shared environment vs genetics in FDRs, but the ORs of approximately 1.5 in husband-wife pairs, while lower than that of FDRs, suggests the effects of shared environment or possibly assortative mating on risk. An increased OR in SDR and TDR support a genetic influence in the development of HT because these relatives are less likely to share common environment (12, 14).

Because nongenetic risks like environment and lifestyle have changed over time, ORs were examined in relatives born in 1974 or earlier and after 1974 to determine if OR has changed over time, with little difference observed (Supplementary Table S11) (26). However, the median age of first HT diagnosis decreased steadily through time (Supplementary Table S12) (26). This could indicate changing environmental factors that result in earlier age of onset. However, the EHR data only date back to 1995, so we do not have access to earlier HT diagnostic records. Therefore, the decreasing age of diagnosis must also reflect individuals born in earlier years who did not have a diagnostic record until later in life. Future studies that assess environmental factors in more detail and how familial risk influences the age of onset will help dissect the complex interaction between genetics and environment in HT risk and help guide screening recommendations.

Though our study predominantly involved white individuals (95% of cases and 90% of controls), we were still able to examine risk across a smaller, but substantial, non-White population. Interestingly, we observed a higher OR among non-White relatives compared to White relatives, which aligns with broader epidemiological trends suggesting potential differences in disease susceptibility across racial groups (23). However, because race and ethnicity in the UPDB are based on self-report and not genetically verified, future research using ancestry-based classification will be crucial for clarifying these findings.

Despite the strengths of our study, including the utilization of a large and well-characterized dataset, several limitations should be acknowledged. The reliance on diagnostic codes for identifying HT cases may introduce misclassification bias, particularly if cases are transient or misdiagnosed as HT when the cause of hypothyroidism is not due to autoimmunity. Additionally, differences in diagnostic practices between clinics may lead to a heterogeneous sample population (30). Furthermore, our study may be subject to ascertainment bias, as individuals with a family history of HT may be more likely to seek medical attention and receive a diagnosis. However, the UPDB minimizes selection and referral bias by accounting for ∼85% of the Utah population across a wide range of health care providers in both urban and rural settings (17). Additionally, the generalizability of our findings may be limited by the demographic characteristics of the study population, which consists of a majority of self-identified White individuals (37). Also, the COVID-19 pandemic in 2020 may have influenced the prevalence and classification of thyroiditis and hypothyroidism, potentially introducing selection bias due to misclassification or disruptions in health care services. COVID-19, which has been linked to autoimmune conditions such as hypothyroidism, was not explicitly accounted for in our analyses (38-40). Further research is needed in diverse populations with genetically verified ancestry to validate our results for broader interpretation.

In conclusion, our study offers valuable insights into the familial risk of HT, emphasizing both genetic and environmental contributions to disease susceptibility. These findings also have clinical implications, as understanding familial clustering of HT can help health care providers identify individuals at higher risk, especially those with affected FDRs. This knowledge could lead to earlier monitoring and intervention, improving disease management. Additionally, knowledge of the genetic influence on HT helps guide follow-up studies, including the identification of specific genetic variants associated with disease susceptibility to potentially inform the development of novel therapeutic approaches.

## Abbreviations

EHR: electronic health record
FDR: first-degree relative
HT: Hashimoto thyroiditis
ICD: International Classification of Diseases
IH: Intermountain Health
IRB: institutional review board
OR: odds ratio
RR: relative risk
SDR: second-degree relative
TDR: third-degree relative
UPDB: Utah Population Database

## References

[1] Hu X, Chen Y, Shen Y, Tian R, Sheng Y, Que H. Global prevalence and epidemiological trends of Hashimoto's thyroiditis in adults: a systematic review and meta-analysis. Front Public Health. 2022;10:1020709. 36311599 10.3389/fpubh.2022.1020709 PMC9608544

[2] Jacobson DL, Gange SJ, Rose NR, Graham NMH. Epidemiology and estimated population burden of selected autoimmune diseases in the United States. Clin Immunol Immunopathol. 1997;84(3):223‐243. 9281381 10.1006/clin.1997.4412

[3] Conrad N, Misra S, Verbakel JY, et al Incidence, prevalence, and co-occurrence of autoimmune disorders over time and by age, sex, and socioeconomic status: a population-based cohort study of 22 million individuals in the UK. Lancet. 2023;401(10391):1878‐1890. 37156255 10.1016/S0140-6736(23)00457-9

[4] Chaker L, Bianco AC, Jonklaas J, Peeters RP. Hypothyroidism. Lancet. 2017;390(10101):1550. 28336049 10.1016/S0140-6736(17)30703-1 PMC6619426

[5] Hussein SMM, AbdElmageed RM. The relationship between type 2 diabetes Mellitus and related thyroid diseases. Cureus. 2021;13(12):e20697. 35106234 10.7759/cureus.20697 PMC8787293

[6] Thvilum M, Brandt F, Almind D, Christensen K, Hegedüs L, Brix TH. Excess mortality in patients diagnosed with hypothyroidism: a nationwide cohort study of singletons and twins. J Clin Endocrinol Metab. 2013;98(3):1069. 23365121 10.1210/jc.2012-3375 PMC3590474

[7] Boelaert K, Newby PR, Simmonds MJ, et al Prevalence and relative risk of other autoimmune diseases in subjects with autoimmune thyroid disease. Am J Med. 2010;123(2):183.e1‐183.e9. 10.1016/j.amjmed.2009.06.030 20103030

[8] Groenewegen KL, Mooij CF, van Trotsenburg ASP. Persisting symptoms in patients with Hashimoto's disease despite normal thyroid hormone levels: does thyroid autoimmunity play a role? A systematic review. J Transl Autoimmun. 2021;4:100101. 34027377 10.1016/j.jtauto.2021.100101 PMC8122172

[9] Ahmed R, Al-Shaikh S, Akhtar M. Hashimoto thyroiditis. Adv Anat Pathol. 2012;19(3):181‐186. 22498583 10.1097/PAP.0b013e3182534868

[10] Brix TH, Kyvik KO, Hegedu¨s L, Hegedu¨s H. A population-based study of chronic autoimmune hypothyroidism in danish twins*. J Clin Endocrinol Metabol. 2000;85:536‐539. 10.1210/jcem.85.2.6385 10690851

[11] Skov J, Calissendorff J, Eriksson D, et al Limited genetic overlap between overt Hashimoto's thyroiditis and graves’ disease in twins: a population-based study. J Clin Endocrinol Metab. 2021;106(4):1101‐1110. 33382429 10.1210/clinem/dgaa956 PMC7993582

[12] Kim HJ, Kazmi SZ, Kang T, et al Familial risk of Hashimoto's thyroiditis among first-degree relatives: a population-based study in Korea. Thyroid. 2021;31(7):1096‐1104. 33514269 10.1089/thy.2020.0213

[13] Villanueva R, Greenberg DA, Davies TF, Tomer Y. Sibling recurrence risk in autoimmune thyroid disease. Thyroid. 2003;13(8):761‐764. 14558919 10.1089/105072503768499653

[14] Thomsen H, Li X, Sundquist K, Sundquist J, Försti A, Hemminki K. Familial risks between Graves disease and Hashimoto thyroiditis and other autoimmune diseases in the population of Sweden. J Transl Autoimmun. 2020;3:100058. 32743538 10.1016/j.jtauto.2020.100058 PMC7388361

[15] Kust D, Matesa N. The impact of familial predisposition on the development of Hashimoto's thyroiditis. Acta Clin Belg. 2020;75(2):104‐108. 30570414 10.1080/17843286.2018.1555115

[16] Dittmar M, Libich C, Brenzel T, Kahaly GJ. Increased familial clustering of autoimmune thyroid diseases. Horm Metab Res. 2011;43((03|3)):200‐204. 21287436 10.1055/s-0031-1271619

[17] Smith KR, Fraser A, Reed DL, et al The Utah population database. A model for linking medical and genealogical records for population health research. Hist Life Course Stud. 2022;12:58‐77.

[18] DuVall SL, Fraser AM, Rowe K, Thomas A, Mineau GP. Evaluation of record linkage between a large healthcare provider and the Utah population database. J Am Med Inform Assoc. 2012;19(e1):e54. 21926112 10.1136/amiajnl-2011-000335 PMC3392872

[19] McLellan T, Jorde LB, Skolnick MH. Genetic distances between the Utah Mormons and related populations. Am J Hum Genet. 1984;36(4):836‐857. 6591796 PMC1684477

[20] Albright FS, Kohlmann W, Neumayer L, et al Population-based relative risks for specific family history constellations of breast cancer. Cancer Causes Control. 2019;30(6):581‐590. 31030355 10.1007/s10552-019-01171-5

[21] Horne BD, Camp NJ, Muhlestein JB, Cannon-Albright LA. Identification of excess clustering of coronary heart diseases among extended pedigrees in a genealogical population database. Am Heart J. 2006;152(2):305‐311. 16875915 10.1016/j.ahj.2005.12.028

[22] Bean LL, May DL, Skolnick M. The mormon historical demography project. Hist Methods. 1978;11(1):45‐53. 11614600 10.1080/01615440.1978.9955216

[23] Smith K, Fraser A. If you link it they will Come, if they like it they will stay: the Utah population database as a model for creating a confidential linked population health research registry. Int J Popul Data Sci. 2018;3(4):60-64.

[24] Jorde LB . Inbreeding in the Utah mormons: an evaluation of estimates based on pedigrees, isonymy, and migration matrices. Ann Hum Genet. 1989;53(4):339‐355. 2624429 10.1111/j.1469-1809.1989.tb01803.x

[25] Wylie JE, Mineau GP. Biomedical databases: protecting privacy and promoting research. Trends Biotechnol. 2003;21(3):113‐116. 12628367 10.1016/S0167-7799(02)00039-2

[26] Bujnis M, DeSalvo, K, Neklason DW, Madsen MJ, Jorde LB. Familial risk of Hashimoto's thyroiditis in a large genealogical database. J Clin Endocrinol Metab. 2025;110(12):e3998‐e4003. 10.1210/clinem/dgaf251 PMC12623010 40290040

[27] Zaletel K, Gaberšček S. Hashimoto's thyroiditis: from genes to the disease. Curr Genomics. 2011;12(8):576. 22654557 10.2174/138920211798120763 PMC3271310

[28] Ragusa F, Fallahi P, Elia G, et al Hashimotos’ thyroiditis: epidemiology, pathogenesis, clinic and therapy. Best Pract Res Clin Endocrinol Metab. 2019;33(6):101367. 31812326 10.1016/j.beem.2019.101367

[29] Mammen JSR, Cappola AR. Autoimmune thyroid disease in women. JAMA. 2021;325(23):2392. 33938930 10.1001/jama.2020.22196 PMC10071442

[30] Garber JR, Cobin RH, Gharib H, et al Clinical practice guidelines for hypothyroidism in adults: cosponsored by the American association of clinical endocrinologists and the American thyroid association. Endocr Pract. 2012;18(6):988‐1028. 23246686 10.4158/EP12280.GL

[31] Chistiakov DA . Immunogenetics of Hashimoto's thyroiditis. J Autoimmune Dis. 2005;2(1):1. 15762980 10.1186/1740-2557-2-1 PMC555850

[32] Lee HJ, Li CW, Hammerstad SS, Stefan M, Tomer Y. Immunogenetics of autoimmune thyroid diseases: a comprehensive review. J Autoimmun. 2015;64:82‐90. 26235382 10.1016/j.jaut.2015.07.009 PMC4628844

[33] Fraser FC . Some underlooked properties of the multifactorial/threshold model. Am J Hum Genet. 1998;62(5):1262‐1265. 9545400 10.1086/301830 PMC1377087

[34] Klubo-Gwiezdzinska J, Wartofsky L. Hashimoto thyroiditis: an evidence-based guide to etiology, diagnosis and treatment. Pol Arch Intern Med. 2022;132(3):16222. 35243857 10.20452/pamw.16222 PMC9478900

[35] Santin AP, Furlanetto TW. Role of estrogen in thyroid function and growth regulation. J Thyroid Res. 2011;2011:875125. 21687614 10.4061/2011/875125 PMC3113168

[36] Krysiak R, Kowalcze K, Okopień B. The effect of testosterone on thyroid autoimmunity in euthyroid men with Hashimoto's thyroiditis and low testosterone levels. J Clin Pharm Ther. 2019;44(5):742‐749. 31183891 10.1111/jcpt.12987

[37] Edelman LS, Guo JW, Fraser A, Beck SL. Linking clinical research data to population databases. Nurs Res. 2013;62(6):438. 24165220 10.1097/NNR.0000000000000002 PMC3907268

[38] Tsivgoulis G, Fragkou PC, Karofylakis E, et al Hypothyroidism is associated with prolonged COVID-19-induced anosmia: a case–control study. J Neurol Neurosurg Psychiatry. 2021;92(8):911–9912. 10.1136/jnnp-2021-326587 33879534

[39] Boelaert K, Edward Visser W, Taylor PN, Moran C, Léger J, Persani L. ENDOCRINOLOGY IN THE TIME OF COVID-19: management of hyperthyroidism and hypothyroidism. Eur J Endocrinol. 2020;183(1):G33‐G39. 32438340 10.1530/EJE-20-0445 PMC7938012

[40] Damara FA, Muchamad GR, Ikhsani R, Hendro, Syafiyah AH, Bashari MH. Thyroid disease and hypothyroidism are associated with poor COVID-19 outcomes: a systematic review, meta-analysis, and meta-regression. Diabetes Metab Syndr. 2021;15(6):102312. 34731819 10.1016/j.dsx.2021.102312 PMC8530797

